# Advancing health technology assessment in the Middle East and North Africa: insights and outcomes from the 2025 HTAi regional meeting

**DOI:** 10.1017/S0266462326103663

**Published:** 2026-03-31

**Authors:** Antonio Migliore, Mouna Jameleddine, Ann Single, Debjani Mueller, Nabil Harzallah, Nicola Vicari, Zakia Ines Harzallah, George Valiotis

**Affiliations:** 1 Health Technology Assessment International (HTAi), Canada; 2 National Authority for Assessment and Accreditation in Healthcare (INEAS), Tunisia; 3 University of Pretoria, Pretoria, South Africa

**Keywords:** health technology assessment, MENA, regional collaboration, universal health coverage, evidence-informed policy

## Abstract

The 2025 Health Technology Assessment International (HTAi) Regional Meeting for the Middle East and North Africa (MENA) was held in Tunis, Tunisia, in September 2025. The meeting, coorganized with the Tunisian National Authority for Assessment and Accreditation in Healthcare (INEAS), focused on advancing equitable, efficient, and innovative health systems through institutionalized HTA practices. Across three core sessions – regional HTA development, system functionality and resilience, and access to medicines – participants shared national experiences, challenges, and collaborative opportunities. Key outcomes emphasized building enabling environments for HTA, capacity development, multi-stakeholder collaboration, and integration of HTA into governance and financing systems. This article highlights lessons learned and identifies strategic recommendations for fostering sustainable HTA growth across the MENA region.

## Introduction

Health systems across the Middle East and North Africa (MENA) face a complex and interrelated set of challenges, including rising healthcare costs, an increasing burden of chronic disease, fragmentation of health system governance, workforce constraints, and, in several settings, political instability and conflict (1). Rapid demographic and epidemiological transitions further amplify pressures on already heterogeneous health systems, increasing the demand for structured, evidence-informed decision-making to ensure efficiency, equity, and resilience. While interest in health technology assessment (HTA) is growing, many countries in the region continue to rely on unstructured or ad hoc evaluation processes (2). HTA is a multidisciplinary process that employs explicit, systematic methods to assess the value of health technologies throughout their lifecycle, thereby supporting the equitable and efficient allocation of limited resources (3). Its increasing prominence in low-, middle-, and high-income settings reflects its relevance for achieving universal health coverage (UHC) and sustainable health system financing (4;5). Across the MENA region, HTA is increasingly recognized as a critical governance tool in response to variable institutional capacity and wide disparities in access to health technologies that make explicit prioritization mechanisms necessary to support equitable coverage decisions and strengthen accountability within health governance structures. In this context, the Health Technology Assessment International (HTAi) MENA Regional Meeting, held in Tunis on 24–25 September 2025 and organized in partnership with the Tunisian National Authority for Assessment and Accreditation in Healthcare (INEAS), provided a neutral platform for dialogue among policymakers, practitioners, academia, industry, and civil society. The event took place at a moment of significant transition, as several MENA countries are moving from exploratory or *ad hoc* HTA activities toward more formalized institutional arrangements. The meeting theme, “*Advancing HTA in the MENA Region: Promoting Equity, Efficiency, and Innovation in Healthcare,”* reflected a shared regional ambition to embed evidence-informed decision-making within health governance structures. Synthesizing insights from this regional dialogue provides a timely perspective on shared challenges, emerging implementation models, and strategic inflection points shaping the next phase of HTA development in the region.

### Meeting structure

The two-day meeting brought together 173 delegates from 28 countries, including representatives from government agencies, HTA bodies, academia, industry, and civil society. Proceedings included a keynote address, three main sessions, and three educational workshops designed to explore the technical, institutional, and political dimensions of HTA development through a collaborative approach. Readers are invited to consult the event agenda, which includes a synopsis of the three workshops and the list of speakers, not included in this article (6).

## Lessons learned for advancing HTA in the MENA region

### HTA institutionalization requires political anchoring and pragmatism

A consistent message across country experiences was that successful HTA implementation depends on more than technical capacity alone. Political commitment, legislative backing, and sustainable financing mechanisms are foundational prerequisites. Countries at different stages of HTA maturity reported that unclear mandates, fragmented legal frameworks, and reliance on *ad hoc* funding undermine continuity and policy relevance. At the same time, participants emphasized the importance of “pragmatic HTA”: flexible, context-sensitive approaches that balance methodological rigor with timeliness and feasibility. International experience shared during the meeting highlighted that overly complex or data-intensive processes risk marginalization if they fail to align with real-world policy cycles. Incremental implementation, starting with priority technologies and progressively expanding scope, was repeatedly identified as a viable pathway for countries with limited resources.

### Capacity building and data infrastructure are persistent bottlenecks

Across the region, shortages of trained human resources and limited access to high-quality local data remain critical constraints. Participants noted gaps in formal HTA education and limited opportunities for applied, on-the-job learning. Weak health information systems, including incomplete registries and underdeveloped electronic health records compound these challenges. The discussions underscored that capacity building must extend beyond analysts to include policymakers, clinicians, and other stakeholders involved in decision-making. Without a shared understanding of HTA principles and limitations, even technically sound assessments may fail to influence policy. Cross-country mentorship, regional training programs, and collaboration with academic institutions were widely viewed as cost-effective strategies to accelerate skills development and methodological consistency.

### Broadening the scope beyond pharmaceuticals is both necessary and complex

While pharmaceuticals remain the dominant focus of HTA activities worldwide, there was a strong consensus that confining assessments solely to medicines significantly restricts the systemic impact of HTA and limits its ability to deliver meaningful health gains across the broader health system. Participants highlighted growing interest in assessing medical devices, diagnostics, digital health solutions, artificial intelligence-based technologies, and public health interventions. However, evaluating nonpharmaceutical technologies presents distinct methodological and organizational challenges. Rapid innovation cycles, limited clinical evidence at launch, and significant organizational implications require adapted frameworks that consider, for instance, safety, usability, lifecycle costs, and system impact. Experiences shared during the meeting reinforced that expanding HTA scope should be gradual and aligned with national priorities and available expertise.

### HTA must be integrated within broader health governance ecosystems

Another recurring lesson was that HTA delivers value only when clearly linked to downstream decisions on pricing, reimbursement, procurement, and benefit design. Participants cautioned against treating HTA as an isolated technical function. Instead, HTA should be embedded within broader governance ecosystems that connect regulation, assessment, purchasing, and service delivery. Discussions on access to medicines further highlighted the need to balance innovation with affordability and financial sustainability. While legislative and resource contexts differ across countries, structural barriers are often similar.

## Strategic recommendations for sustainable HTA growth in MENA

Drawing on these lessons, several strategic priorities emerge for fostering sustainable HTA development across the region:

- Strengthen institutionalization and governance – Establish clear legal mandates, independent governance structures with dedicated resources, and predictable financing mechanisms that anchor HTA within national decision-making processes.
- Invest in comprehensive capacity building – Develop coordinated education and training strategies that target analysts, policymakers, clinicians, and other stakeholders, combining formal education with practical, mentored experience.
- Improve data infrastructure and access – Strengthen health information systems, registries, and data-sharing mechanisms to support locally relevant assessments and reduce reliance on external evidence alone.
- Promote inclusive stakeholder engagement – Engage patients, clinicians, industry, and policymakers early and transparently to improve the relevance, acceptability, and uptake of HTA recommendations.
- Encourage regional collaboration and alignment – Leverage cross-country cooperation, shared methodological guidance, and joint training initiatives to reduce duplication of effort and accelerate learning across different HTA maturity levels.
- Capitalize on the outputs of the European Joint Clinical Assessments to inform HTA in the MENA region, to reduce duplication of effort, enhancing process efficiency, and building systematically on evidence and assessments already generated in other jurisdictions.
- Integrate HTA into policy and financing decisions – Explicitly link HTA outputs to procurement, reimbursement, and benefit-package design to ensure assessments translate into tangible system-level impact.

## Discussion and conclusions

While many of the challenges identified during the meeting mirror those observed in other emerging HTA settings, discussions highlighted several constraints that are particularly salient in the MENA context. These included fragmented healthcare systems, weak data infrastructures, noninteroperable data systems across payers and providers, limited transparency regarding decision-making mechanisms, and heavy reliance on coverage and pricing decisions from other contexts. Participants emphasized that these factors complicate the conduct of HTA and the full transition to evidence-informed priority setting, underscoring the need for regionally adapted approaches rather than direct transplantation of models from other settings. The discussions during the event echoed a pattern now visible across multiple MENA settings: institutionalization succeeds when countries pair clear mandates with pragmatic, stepwise methods, consistent stakeholder engagement, and deliberate capacity building. Recent work in the United Arab Emirates reached nearly identical conclusions, calling for a nationally coordinated HTA function, ring-fenced public financing for appraisals, expansion of graduate and postgraduate training, and a widening of the remit beyond pharmaceuticals to include devices and public-health interventions (7). Egypt’s reform pathway likewise shows that legal scaffolding alone is not sufficient: early investments in awareness, skills, and transparent processes are needed to translate statutory intent into routine practice and to build trust among decision-makers and market actors (8). Algeria’s roadmap adds further weight to these points, recommending a tailored implementation plan grounded in local resources, governance realities, and an explicit education strategy to make HTA outputs policy-relevant and timely (9). Experience from middle-income settings shows that the earliest and most tangible returns of HTA are not necessarily immediate budgetary savings or health gains, but stronger governance: clearer decision rules, greater transparency, and enhanced accountability. Over time, these institutional improvements create the conditions for more sustainable fiscal and health outcomes (10). Country practice illustrates how this evolution occurs in reality. In Tunisia, HTA of innovative medicines is undertaken to guide uptake and coverage decisions and facilitate the move toward value-based pricing arrangements. This experience highlights how structured, methodologically robust evaluations can be embedded within procurement and coverage processes, ensuring that resource allocation is grounded in explicit value considerations rather than implicit or *ad hoc* criteria (11). In the UAE, recent work to develop locally appropriate cost-effectiveness thresholds and decision tools shows how methods can be adapted to national preferences while remaining anchored in international standards (12). In Oman, the adoption of formal HTA methodological guidelines illustrates how international standards can be embedded within national decision-making. By linking clinical value, cost–utility analysis, budget impact assessment, and transparency requirements, the framework supports more consistent and evidence-based coverage decisions (13) while the first national health economic evaluation guideline to standardize methods and support evidence-based healthcare decisions have recently been released in Lebanon, with the aim to improve transparency and resource allocation (14). These experiences mirror the meeting’s emphasis on HTA frameworks where processes are rigorous yet proportionate, fast enough for policy windows, and connected to budgeting and benefit-design levers. Against this backdrop, participants perceived HTAi as uniquely positioned to accelerate the next phase. First, as a neutral convener, HTAi can sustain a safe, multi-stakeholder dialogue that keeps political and technical actors aligned during the “messy middle” of implementation – where mandates are interpreted, methods localized, and trade-offs managed. Second, HTAi’s Interest Groups (15) and Policy Forums can operationalize peer-to-peer mentorship across the region, pairing mature programs with emerging ones to codevelop topic-specific methods and to share submission templates, appraisal checklists, and deliberative processes. Finally, by connecting MENA actors to global conversations on value frameworks and lifecycle HTA, HTAi can help ensure regional methods keep pace with evolving practices while remaining context-sensitive. In sum, the Tunis meeting did more than catalogue needs; it clarified a shared playbook now visible across the MENA region: build capacity, codify transparent methods, broaden scope, and tie HTA outputs to procurement and reimbursement. HTAi is continuing this discussion with HTA experts from the different countries in the format of a Policy Dialogue, which is intended to underscore the significance of collaboration and cocreation among stakeholders, providing a “safe space” that encourages participants to discuss and subsequently collaborate to ensure that HTA is a fully functional and integral component of their healthcare decision-making (16). The output of the HTAi Policy Dialogue for MENA will be the focus of another publication, which is currently in preparation.

## Limitations

This perspective draws on synthesized insights from a regional meeting and does not claim to represent consensus views of all participants or countries in the MENA region. Some countries were not represented, and variation in HTA maturity may limit generalizability. Nonetheless, the lessons and recommendations outlined here provide a qualitative foundation to inform strategic planning and dialogue on HTA institutionalization across the region.

## References

[1] World Health Organization, Regional Office for the Eastern Mediterranean. Health and well-being profile of the eastern Mediterranean region: An overview of the health situation in the region and its countries in 2023. Cairo: WHO EMRO; 2024. Available from: https://applications.emro.who.int/docs/9789292744656-eng.pdf (accessed October 24, 2025).

[2] Mirelman AJ, Goel K, Edejer TT. The global landscape of country-level health technology assessment processes: A survey among 104 countries. Health Policy Open. 2025;8:100138. 40236923 10.1016/j.hpopen.2025.100138 PMC11999493

[3] O’Rourke B, Oortwijn W, Schuller T. The new definition of health technology assessment. Int J Technol Assess Health Care. 2020;36(3):187–190. 32398176 10.1017/S0266462320000215

[4] Guzman J, Fan VY, Baker P. The future of health technology assessment in low- and middle-income countries. Health Syst Reform. 2023;9(3):2400399. 39466901 10.1080/23288604.2024.2400399

[5] WHO Regional Office for the Eastern Mediterranean. Building resilient health systems towards universal health coverage and health security in the Eastern Mediterranean Region. Technical paper EM/RC69/4; 2022. Available from: https://applications.emro.who.int/docs/Build-resilient-health-systems-UHC-EMR-eng.pdf (accessed October 24, 2025).

[6] Health Technology Assessment International. HTAi Middle East and North Africa Regional Meeting. Available from: https://htai.org/event/htai-middle-east-and-north-africa-regional-meeting/ (accessed November 4, 2025).

[7] Alnaqbi KA, Elshamy AM, Gebran N, et al. Consensus-based recommendations for the implementation of health technology assessment in the United Arab Emirates. Value Health Reg Issues. 2024;43:101012. 38861786 10.1016/j.vhri.2024.101012

[8] Pinilla-Domínguez P, Chalkidou K, Akeel A, et al. Institutionalizing health technology assessment in Egypt: Situational analysis and roadmap. Front Pharmacol. 2022;13:1014658. 36438785 10.3389/fphar.2022.1014658 PMC9682258

[9] Hedibel M, Ghanassi F-Z, El-Fass KA, et al. Designing a roadmap for health technology assessment implementation in Algeria. Cureus. 2024;16(7):e65558. 10.7759/cureus.65558. 39192895 PMC11349251

[10] Fasseeh AN, Elezbawy B, Gamal M, et al. Impact of health technology assessment implementation with special focus on middle-income countries. Health Policy Technol. 2022;11(4):100688.

[11] Jameleddine M, Harzallah N, Grati H, et al. INEAS’S cost-effectiveness analysis of vemurafenib: Paving the way for value-based pricing in Tunisia. J Mark Access Health Policy. 2024;12(4):294–305. 39464179 10.3390/jmahp12040023 PMC11503406

[12] Aldallal S, Alnaqbi KA, Elshamy AM, et al. Thresholds for the value judgement of health technologies in the United Arab Emirates: A consensus approach through voting sessions. BMJ Open. 2024;14(11):e090344. 10.1136/bmjopen-2024-090344 PMC11535693 39496369

[13] Wani S, Alsabti H, Allamki S, et al. Methodological guidelines for health technology assessment in Oman. J Pharm Policy Pract. 2025;18(1):2596523. 41384030 10.1080/20523211.2025.2596523 PMC12690758

[14] Daccache C, Rizk R, Hiligsmann M, Evers SMAA, Karam R. The Lebanese health economic evaluation guideline. Expert Rev Pharmacoecon Outcomes Res. 2025;25(4):551–565. 39772975 10.1080/14737167.2025.2450322

[15] Migliore A, Vicari N, Valiotis G, Single A. Connecting minds and catalyzing collaboration: The interest groups of health technology assessment international. Int J Technol Assess Health Care. 2025;41(1):e43. 40619817 10.1017/S0266462325100287 PMC12303693

[16] Migliore A, Vicari N, Turk E, Sucu R. Driving policy dialogue on health technology assessment in Eastern Europe and Central Asia: Reporting from an initiative of health technology assessment international. Int J Technol Assess Health Care. 2025;41(1):e12. 39910984 10.1017/S0266462325000066 PMC11894386

